# Do popular media such as movies aggravate the stigma of mental disorders?

**DOI:** 10.4103/0019-5545.33267

**Published:** 2007

**Authors:** Harshal Pandve, A. Banerjee

**Affiliations:** Dept. of Community Med, D. Y. Patil Medical College, Pune - 18, India. E-mail: amitavb@gmail.com

Sir,

Mental and behavioral disorders are found in all regions, countries and societies.[1]

Hollywood films frequently handle psychiatric themes. To mention few, “One flew over the cuckoo's nest,” “A Beautiful Mind,” “Mr. Jones,” “As Good As it Gets,” “Psycho,” and “The English Patient.” All these have been handled with a touch of professionalism.

On the other hand, Indian films usually portray mental disorders in the form of crude comedy, showing the victim of mental illness as a subject of ridicule. This practice aggravates the stigma associated with mental disorders. Few serious efforts have been made to handle the issue of mental illness in a scientific and realistic manner. Way back in 1960s/70s, the films “Khamoshi” and “Khilona” attempted to portray mental disorders with some seriousness albeit leaving much to be desired in scientific content. Now the scene is changing—recently, films like “Maine Gandhi Ko Nahi Mara” (2006) and “Woh Lamhe” (2006), based on psychiatric disorders have been handled with some degree of scientific credibility.

“Maine Gandhi Ko Nahi Mara”, directed by Jhanu Barua, deals with Alzheimer's disease, an issue that hasn't been tackled on Hindi screen before. It throws light on the lives of patients with such a disease—how the patient himself and his family suffer due to his mental condition. It also shows society's unaccepting and hypocritical attitude towards members of such families. People still think that mental illness runs in families and many young women face such problems before and during their marriage especially in Indian society where arranged marriages are still the norm. It also shows the parent-child relationship, the feeling of being ‘unwanted’ that a senior citizen experiences at times and the caregivers' burden.[2] The film “Woh Lamhe” was based on the life of the late actress Parveen Babi, who suffered from schizophrenia.

Some of the films in regional languages are also based on psychiatric disorders. In the Marathi film known as “Devrai” which is based on psychiatric disorders like schizophrenia, most issues associated with severe and persistent mental illness are addressed. Risk factors such as faulty parenting, unhealthy family life and their correlation with the patient's chemical imbalance are tackled well. Other issues like the patient's capacity to resist giving in to delusions and hallucinatory experience as well as family involvement are all explored in some detail. The film depicts the internal experience and logic of hallucinations in an in-depth and sensitive manner. It depicts the social and familial stigma surrounding mental illness as well as the concerns and dilemmas faced by caregivers. It also tries to explore family insights, support structures and alternative institutions of mental health care.[3] Another Marathi film, “Ratra Arambh” is also based on schizophrenia. The Malayalam film, “Thanmatra” shows dementia affecting a middle-aged person and focuses attention on the social difficulties of dealing with dementia.[4]. The film shows the ensuing disruption of family life. The doctor who counsels the family assures them that the patient is not suffering because he is not aware of the condition. There is no structured social support in India to help a family cope with such a situation. The film is noteworthy for tackling a difficult theme.

Film media should be used to advantage to dispel the stigma associated with psychiatric disorders. Care should be taken to ensure that only scientifically sound messages are conveyed to the lay audience. The help of the censor board should be taken to check the dissemination of erroneous messages related to mental disorders, which would only aggravate the traditional stigma associated with such disorders. Intellectuals like Dr. Mohan Agashe, an influential movie personality as well as a renowned psychiatrist, can play an active role towards this goal.

## References

[1] Park K (2007). Mental Heath. Textbook of preventive and social medicine.

[2] Praharaj SK, Sinha VK, Arora M (2006). Movie review “Maine Gandhi Ko Nahi Mara (I Have Not Killed Gandhi). Indian J Soc Psychiatry.

[3] Dandekar D (2005). Devrai: Sensitive but from the caregiver's point of view. Indian J Med Ethics.

[4] Thomas G (2006). Coping with dementia: Thanmatra. Indian J Med Ethics.

